# Experimental Study of Liquid and Gas Gate Valve Internal Leakage Testing Based on Ultrasonic Signal

**DOI:** 10.3390/s25185909

**Published:** 2025-09-21

**Authors:** Tingwei Wang, Xinjia Ma, Shiqiang Zhang, Qiang Feng, Xiaomei Xiang, Hui Xia

**Affiliations:** 1 Sichuan University Pittsburgh Institute, Chengdu 610207, China; 2022141520227@stu.scu.edu.cn; 2 School of Mechanical Engineering, Sichuan University, Chengdu 610065, China; 2023141410421@stu.scu.edu.cn (X.M.); 2023141410264@stu.scu.edu.cn (S.Z.); xxm@scu.edu.cn (X.X.); 3 CNPC Chuanqing Drilling Engineering Co., Ltd., Safety, Environmental Protection and Quality Supervision Institute, Chengdu 610051, China; ktfq@cnpc.com.cn

**Keywords:** high-pressure liquid and gas valve, internal leakage quantification, ultrasonic signal, acoustic wave characteristics

## Abstract

This study presents an experimental analysis of high-pressure liquid and gas gate valve leakage under multiple operating conditions, based on the variation patterns of ultrasonic signals. Focusing on a multi-physics field analysis of gate valve internal leakage and corresponding experiments, this research illustrates the acoustic wave characteristics of gate valves across diverse working media, pressures, internal leakage defect sizes, and valve diameters. By drawing upon both fluid mechanics and acoustics theory, an analytical approach suited to high-pressure gate valve leakage issues is devised. Separate high-pressure gate valve leakage test platforms for liquid and gas environments were designed and constructed, enabling 126 groups of tests under varying conditions, which include one measurement per condition of the valve size, defect size, and pressure value. These experiments examine the quantitative correlation of internal leakage flow rates and ultrasonic signal measurements under different situations. In addition, the distinct behaviors and principles exhibited by high-pressure liquid gate valves and gas gate valves are compared. The findings provide theoretical and technical support for quantifying high-pressure gate valve leakage. The study analyzes the theoretical basis for the generation of ultrasonic signals from valve internal leakage, providing specific experimental data under various operating conditions. It explains the various observations during the experiments and their principles. The conclusions of this research have practical engineering value and provide important references for future studies.

## 1. Introduction

Valves are core components in fluid control systems by governing flow interruption, pressure regulation, and flow-path distribution [1,2,3,4]. They are extensively applied in industrial facilities such as storage tanks and pipelines. Internal leakage is especially critical among the various failure modes of high-pressure gate valves, since fluid continues to pass through even after the valve is closed. This leakage can lead to substantial risks, making accurate and rapid internal leakage detection—particularly in quantitative terms—a major concern in industrial operations [5,6,7,8].

Current valve leakage detection techniques include nitrogen injection, vibration analysis, infrared thermography, negative-pressure wave monitoring and acoustic emission. However, these methods often fail to achieve high accuracy and may not accommodate high-pressure, multi-medium situations [7,8,9,10,11,12]. By contrast, ultrasonic detection focuses on the transient ultrasonic signals generated when high-velocity flows pass through defects. Owing to the rapid propagation speed and robust penetration ability of ultrasonic waves, this method effectively resists signal attenuation in complex flow channels, through time-frequency analysis, leakage signals can be distinguished from background noise with good sensitivity and interference resistance, providing a viable solution for quantifying internal leakage of gate valves under high-pressure conditions [13,14,15,16,17]. Existing research on ultrasonic detection of internal valve leakage demonstrates a growing trend of integrating multiple technical approaches [18,19,20,21,22,23]. By establishing a mathematical model that connects acoustic signal features with leakage parameters, non-invasive online monitoring and predictive capabilities can be gradually achieved. However, precise quantification and standardized testing for high-pressure and broad-range media remain future challenges [24,25,26,27,28,29]. Providing specific experimental data under various operating conditions greatly aids the development of mathematical models and the verification of related theories [30,31,32,33]. Therefore, it is highly necessary to summarize a quantitative theory of high-pressure gate valve internal leakage based on ultrasonic measurements, grounded in concrete experimental findings.

In order to address the quantitative leakage requirements of high-pressure gate valves handling liquids and gases, this study focuses on valves operating above 10 MPa. Under various pressures, defect sizes, and valve diameters, water and air were selected as the test media. First, the principles of ultrasonic detection and the source mechanisms of gate valve leakage were investigated, primarily in flow fields and acoustic fields. Subsequently, an experimental platform for high-pressure gate valve leakage was developed to accommodate both liquid and gas conditions. Experiments were conducted on three valve sizes (DN65, DN78, DN130), three defect sizes (0.1 mm, 0.2 mm, 0.3 mm), two working media (liquid and gas), and pressures (10 MPa–70 MPa, each 5 Mpa increment served as a teat point). The nominal diameters for the valves, the choices of fluid and gas media, and the pressure levels in our experiments are all consistent with common industrial practices, thus broadening the applicability of our results and offering theoretical and experimental support for leak detection in factories. We selected 0.1 mm as our smallest defect size to match the finest precision achievable under current experimental conditions, ensuring that our investigation captures details at the highest possible resolution. Those experiments include one measurement per condition of the valve size, defect size, and pressure value. Finally, based on theoretical and experimental findings, a comparative analysis was performed, elucidating the acoustic theory and characteristics of high-pressure gate valve leakage in both liquid and gas environments, and summarizing the influence of different operating factors on ultrasonic signals associated with internal leakage.

## 2. Theoretical Analysis

### 2.1. Mechanism of Noise Generation Due to Internal Leakage in Valves

The noise mechanism induced by internal valve leakage is primarily tied to the fluid’s dynamic behavior and its interaction with the valve structure. Due to operational wear or erosion caused by the working medium, the seal between the valve seat and the gate may fail. When a large pressure difference exists between the upstream and downstream sides, fluid accelerates sharply as it passes through the leakage gap, forming turbulence and vortices at the leakage site, which then radiate sound. Although several noise sources can arise from internal leakage, the dominant source is jet noise. We use the Navier–Stokes equation in Equation (1) to describe

(1) $$\left\{\begin{array}{c} \frac{\partial u_{i}}{\partial x_{i}}=0\\ \frac{\partial u_{i}}{\partial t}+u_{j}\frac{\partial u_{i}}{\partial x_{j}}=-\frac{1}{\rho }\frac{\partial p}{\partial x_{i}}+v\frac{\partial^{2}u_{i}}{\partial x_{j}\partial x_{j}}+S_{i} \end{array}\right.$$

where $u_{i}$ is the velocity component, *p* is the pressure, *ν* is the kinematic viscosity, and $S_{i}$ is the source term.

In this study, the numerical simulation of gate valve internal leakage is conducted in two main parts: flow field numerical simulation and acoustic field numerical simulation [34,35,36,37].

### 2.2. Theoretical Basis of Flow Field

In the flow field simulation section of this study, we employ the Scale-Adaptive Simulation (SAS) model [37,38,39,40], which is derived from the Unsteady Reynolds-Averaged Navier–Stokes (URANS) approach. By introducing turbulence scale transport theory and constructing an exact transport equation for the product of turbulent kinetic energy and length scale (kL), this model directly captures the influence of large-scale vortices on the mean flow. It is incorporated into the k-ω (SST) framework, as shown in Equation (2). Specifically, an SAS source term $Q_{SAS}$ is introduced into the transport equation for the turbulent vorticity ω, constituting a critical improvement over traditional RANS models.

(2) $$\frac{\partial \rho \omega }{\partial t}+\frac{\partial }{\partial x_{i}}\left(\rho u_{i}\omega \right)=\alpha \frac{\omega }{k}G_{k}-\rho \beta \omega^{2}+Q_{SAS}+\frac{\partial }{\partial x_{i}}\left[\left(\mu +\frac{\mu_{t}}{\sigma_{\omega }}\right)\frac{\partial \omega }{\partial x_{j}}\right]\;+\left(1-F_{1}\right)\frac{2\rho }{\sigma_{\omega ,2}}\frac{1}{\omega }\frac{\partial k}{\partial x_{j}}\frac{\partial \omega }{\partial x_{j}}\;$$

$Q_{SAS}$ source term is introduced through a dynamic coupling between the von Kármán length scale ($L_{vK}$) and the grid cell size Δ, which achieves the self-adaptive regulation of the turbulence scale:

(3) $$Q_{SAS}=max\left[\rho \eta_{2}kS^{2}{\left(\frac{L}{L_{vK}}\right)}^{2}-C\cdot \frac{2\rho k}{\sigma_{\phi }}max\left(\frac{1}{\omega^{2}}\frac{\partial \omega }{\partial x_{j}}\frac{\partial \omega }{\partial x_{j}},\frac{1}{k^{2}}\frac{\partial k}{\partial x_{j}}\frac{\partial k}{\partial x_{j}}\right),0\right]$$

the turbulence length scale *L*,

(4) $$L=\frac{\sqrt{k}}{c_{\mu }^{1/4}\cdot \omega }$$

and the von Kármán length scale $L_{vK}$, where Δ is the characteristic size of the grid cell):

(5) $$L_{vK}=k|\frac{U^{\prime }}{U^{\prime \prime }}|$$

This approach enables the model to retain the computational efficiency of traditional RANS in stable flow regions, while automatically activating LES-like resolution in unstable flow regions by damping high-wavenumber fluctuations. In doing so, it achieves a balance between computational accuracy and efficiency in the study of internal leakage evaluation for high-pressure liquid/gas gate valves. Those model parameters like $\eta_{2}$= 3.51, $\sigma_{\phi }$= 2/3, *C* = 2 are calibrated based on classical boundary-layer theory to ensure a broadly applicable description of complex flow fields.

### 2.3. Theoretical Basis of Acoustic Field

In this study, we choose the Ffowcs Williams–Hawkings (FW-H) equation method [38], and the acoustic propagation equation is provided as shown in Equation (6):

(6) $${□}^{2}\left[\left(\rho -\rho_{0}\right)c^{2}H\left(f\right)\right]=\frac{\partial^{2}}{\partial_{xi}\partial_{xj}}\left[T_{ij}H\left(f\right)\right]-\frac{\partial }{\partial_{xi}}\left[L_{i}\delta \left(f\right)\right]+\frac{\partial }{\partial_{t}}\left[Q\delta \left(f\right)\right]$$

where

(7) $$T_{ij}=pu_{i}u_{j}+\left(p^{\prime }-c^{2}p^{\prime }\right)\delta_{ij}-\tau_{ij}$$

In Equation (6), there are three source terms: quadrupole noise sources, (dipole sources) load noise sources, and (monopole sources) thickness noise sources. The thickness and load noise sources are surface distributions of the source terms, as indicated by *δ*(*f*). When an object is enclosed within the control surface, the thickness noise source term represents the noise generated by fluid displacement caused by the object’s motion, and the load noise source term represents the noise caused by unsteady aerodynamic loads exerted by the fluid on the object. Additionally, the quadrupole noise source represents a volume source distribution, as shown by *H*(*f*), which signifies noise sources resulting from nonlinearity, primarily caused by vortex disturbances, shock waves, local changes in sound speed, and similar factors.

## 3. Experiment Process and Results Analysis

This gate valve leakage tests were conducted under high-pressure ranges of 10 MPa to 70 MPa, using both liquid and gas media which include one measurement per condition of the valve size, defect size, and pressure value.

**Figure 1:**
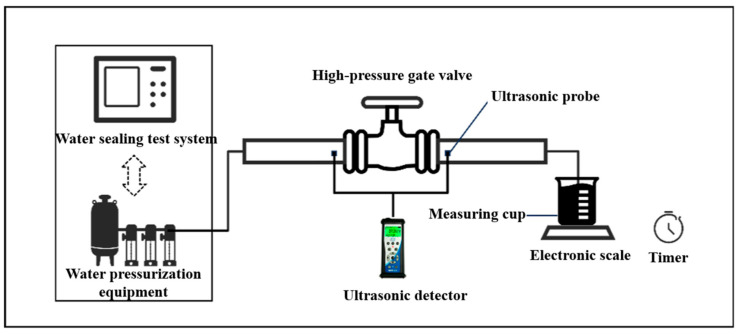
Design drawing of the experimental platform for internal leakage of high-pressure liquid gate valves.

We construct two high-pressure gate valve leakage test platforms for liquid and gas. The design of liquid test platform drawings is shown in Figure 1, respectively. The liquid high-pressure gate valve leakage test platform includes a water-sealing high-pressure test system, a high-pressure gate valve, an ultrasonic detector, ultrasonic probes, a 10 L measuring cup, an electronic scale, and a timer. We employed a high-pressure water sealing test system, which achieves precise control in the conventional pressure range of 0–100 MPa. It integrates an industrial-grade 4 U computer and a Siemens programmable logic controller (PLC), along with a 0.25 FS% high-accuracy pressure transmitter and an XST digital display meter. By utilizing frequency conversion and servo motor control, the system can attain a pressure measurement accuracy of 0.5–1%. We also use SDT200 ultrasonic detector, which works rely on a highly sensitive ultrasonic sensor (0–100 kHz broadband response) to capture the high-frequency acoustic waves generated by internal fluid leakage within the valve, converting them into audible signals and visual electrical signals. For the test chamber, the inlet of the gate valve under examination is connected to the pressurized pipeline of the water-sealing high-pressure test system, and a measuring cup is placed beneath the outlet to collect outputs. To reduce splashing, another measuring cup is mounted at the valve outlet. During pressurization, no personnel are permitted in the chamber; therefore, the measuring cup at the valve outlet (which collects the leaked fluid) is positioned on an electronic scale, and its readings are recorded remotely via a camera to track leakage over a specific duration. Two RS2 NL contact-type ultrasonic sensors are positioned symmetrically at the valve inlet and outlet, with their wiring connected to an SDT200 ultrasonic detector located outside the chamber.

**Figure 2:**
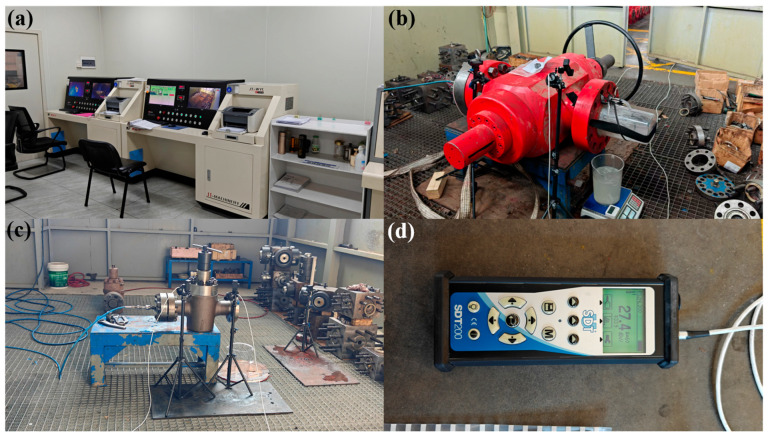
Experimental Platform for Internal Leakage of High-pressure Liquid Gate Valves: (**a**) the water-sealing test system; (**b**) a photograph of the DN130-PN105 valve; (**c**) the DN65-PN70 valve; and (**d**) the SDT200 ultrasonic detection instrument.

During testing, operators leave the chamber and close the door. The water-sealing high-pressure test system is then used to pressurize the valve. Once the valve pressure is stabilized at the target level, the ultrasonic detector is used to measure the dBμV RMS values of the RS2 NL sensors at both inlet and outlet (three readings each, averaged). Simultaneously, variations in the electronic scale readings are observed remotely via the camera, recording the mass of leaked fluid over one minute to determine the leakage flow rate, and documented and analyzed the measurement. The experimental devices are shown in Figure 2. Figure 2a shows the water-sealing test system; Figure 2b is a photograph of the DN130-PN105 valve; Figure 2c indicates the DN65-PN70 valve; and Figure 2d is the SDT200 ultrasonic detection instrument.

**Figure 3:**
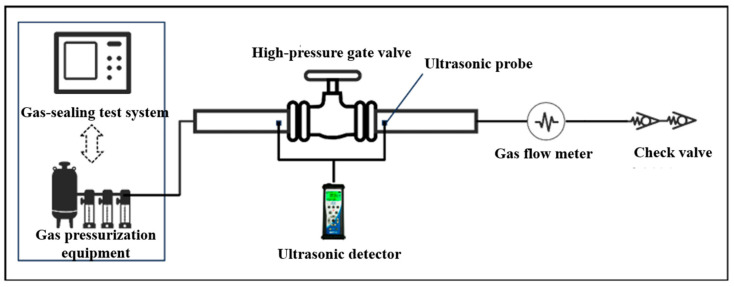
Design drawing of the experimental platform for internal leakage of high-pressure gas gate valves.

**Figure 4:**
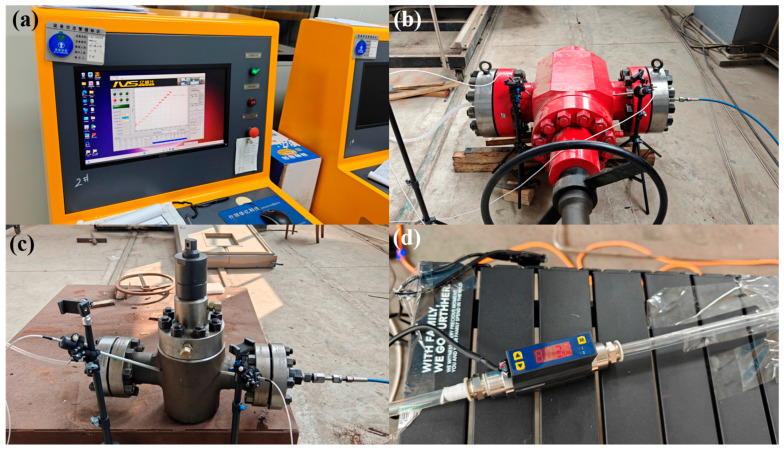
Experimental platform for internal leakage of high-pressure gas gate valves: (**a**) the gas-sealing test system; (**b**) a photograph of the DN130-PN105 valve; (**c**) the DN65-PN70 valve; and (**d**) the MF3000M-1500-R-BAN-A gas flow meter.

The design of liquid test platform drawings is shown in Figure 3, respectively. The gas high-pressure gate valve leakage test platform primarily consists of a gas-sealing test system, gas pressurization equipment, an ultrasonic detector, ultrasonic probes, a high-pressure gate valve, gas pipelines, a gas volumetric flow meter, and a check valve. During gas-pressure testing, the gas-sealing high-pressure system is operated to pressurize the valve. After adjusting and monitoring the valve pressure until it remains steady at the set value, the dBμV RMS readings of the RS2 NL contact sensors at the inlet and outlet are obtained from the ultrasonic detector (three measurements each, averaged). Concurrently, the gas volumetric flow meter is used to measure the leakage flow rate, and the test data are recorded and processed accordingly. The experimental devices are shown in Figure 4. Figure 4a shows the gas-sealing test system; Figure 4b is a photograph of the DN130-PN105 valve; Figure 4c indicates the DN65-PN70 valve; and Figure 4d is the MF3000M-1500-R-BAN-A gas flow meter.

**Figure 5:**
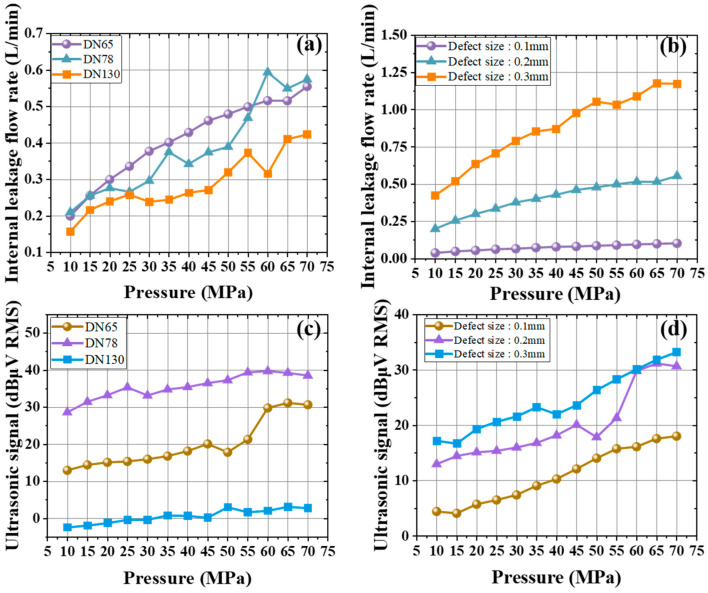
The variation in the internal leakage flow rate of the gate valve in liquid under different pressures with (**a**) different diameters with 0.2 mm defect size and (**b**) different defect sizes. The change in the ultrasonic signal at the outlet of the gate valve in liquid due to internal leakage with the pressure with (**c**) different diameters with 0.2 mm defect size and (**d**) different defect sizes.

Figure 5a,b shows the internal leakage flow rate under different pressures. When the leakage defect size is 0.2 mm and the pressure ranges from 10 MPa to 70 MPa, Figure 5a shows how the flow rate changes for three valve diameters. For the 65 mm, 78 mm, and 130 mm valves, the flow rate generally rises as pressure increases. At a pressure of 65 MPa, the flow rates of the 65 mm and 78 mm valves differ by only about 0.2 L/min. For the 78 mm and 130 mm valves at 25 MPa, 35 MPa, 45 MPa, and 60 MPa, their flow rates move in opposite directions. This indicates that a larger valve diameter is more sensitive to pressure, resulting in certain fluctuations in the curve. For the 65 mm valve within a pressure range of 10 MPa to 70 MPa under three defect sizes, Figure 5b shows that as pressure rises, the leakage flow rate increases steadily for defect sizes of 0.1 mm, 0.2 mm, and 0.3 mm. However, the rate of increase differs: a larger defect size leads to a faster growth in flow rate.

Figure 5c,d examines the ultrasonic signals associated with internal leakage at different pressures. When the defect size is 0.2 mm and the pressure ranges from 10 MPa to 70 MPa, Figure 5c shows that the outlet ultrasonic signal increases gradually for all three diameters. The 78 mm and 130 mm valves exhibit a rise of about 10 dBμV RMS. Compared to these moderate changes, the 65 mm valve shows a sudden surge in its ultrasonic signal between 50 MPa and 60 MPa. After about 60 MPa, the outlet signals for all three diameters tend to stabilize. When the 65 mm valve operates between 10 MPa and 70 MPa, Figure 5d shows that the outlet ultrasonic signal grows as pressure increases, regardless of defect size. The 0.1 mm and 0.3 mm defects show similar curve in ultrasonic signal. However, at the 0.2 mm defect size, the signal increases steadily up to about 40 MPa and then fluctuates more strongly beyond 60 MPa. Under the same pressure, valves with larger diameters and smaller defect cross-sectional areas exhibit lower dBuV RMS values at the outlet.

**Figure 6:**
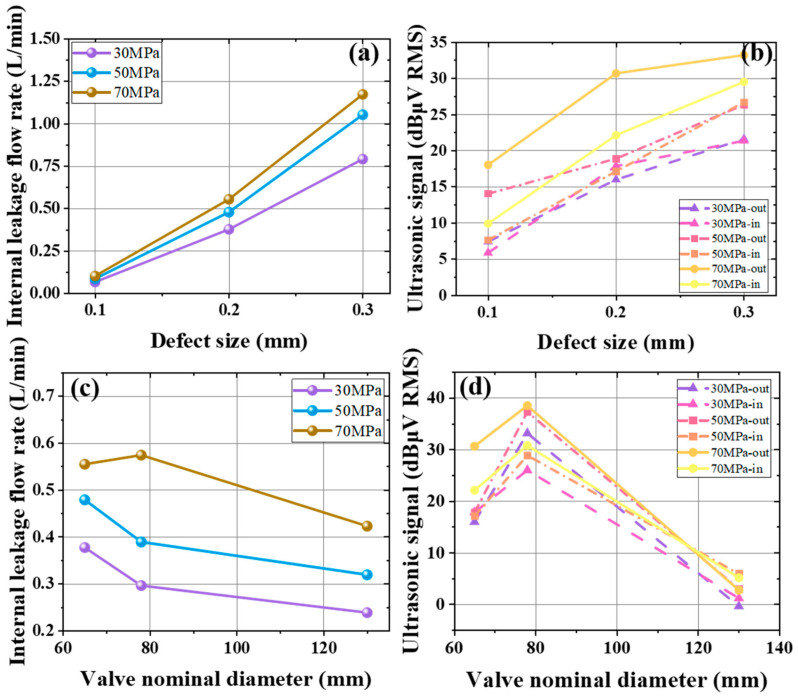
(**a**) Variation in the internal leakage flow rate of the gate valve when the valve diameter is 65 mm in liquid under different defect sizes. (**b**) Variation in the ultrasonic signals at the inlet and outlet of the gate valve when the valve diameter is 65 mm in liquid under different defect sizes. (**c**) Variation in the internal leakage flow rate of the gate valve in liquid under different diameters. (**d**) Variation in the ultrasonic signals at the inlet and outlet of the gate valve for a 0.2 mm defect in liquid under different diameters.

Figure 6a illustrates the internal leakage flow rate under different defect sizes. When the valve diameter is 65 mm and the defect size ranges from 0.1 mm to 0.3 mm, the flow rate at pressures of 30 MPa, 50 MPa, and 70 MPa is shown. Under all three pressures, the internal leakage flow rate increases linearly with defect size, and higher pressures yield larger increases.

Figure 6b presents the ultrasonic signal analysis for varying defect sizes. With a 65 mm valve diameter at three pressures, Figure 6b compares inlet and outlet ultrasonic signals increases as defect size grows. Higher pressures and larger defects lead to higher signal values, reaching a maximum of 33.4 dBμV RMS at a 0.3 mm defect and 70 MPa pressure. The signals rise with defect size, and the outlet signal always exceeds the inlet. It shows that leakage-induced ultrasonic features concentrate at the outlet, making it a more effective monitoring location.

For different valve diameters, Figure 6c shows the internal leakage flow rate when the defect size is 0.2 mm and diameters range from 65 mm to 130 mm at three pressures. Generally, the leakage flow decreases as diameter increases. However, for a 78 mm valve at 70 MPa, the flow rate is markedly higher than other diameter–pressure cases. Under these conditions, the overall trend is that smaller diameters and higher pressures result in higher flow.

Figure 6d examines the ultrasonic signal under various valve diameters for a 0.2 mm defect. With diameters from 65 mm to 130 mm at three pressures, Figure 6d compares inlet and outlet signals. Both grow and then diminish as diameter increases. At 65 mm, signals at 30 MPa and 50 MPa are similar, but at 70 MPa the outlet signal clearly surpasses the inlet. For the 78 mm valve, outlet signals are consistently higher than inlet signals across all pressures, whereas for the 130 mm valve, the inlet signal is always higher than the outlet under the three pressure levels.

**Figure 7:**
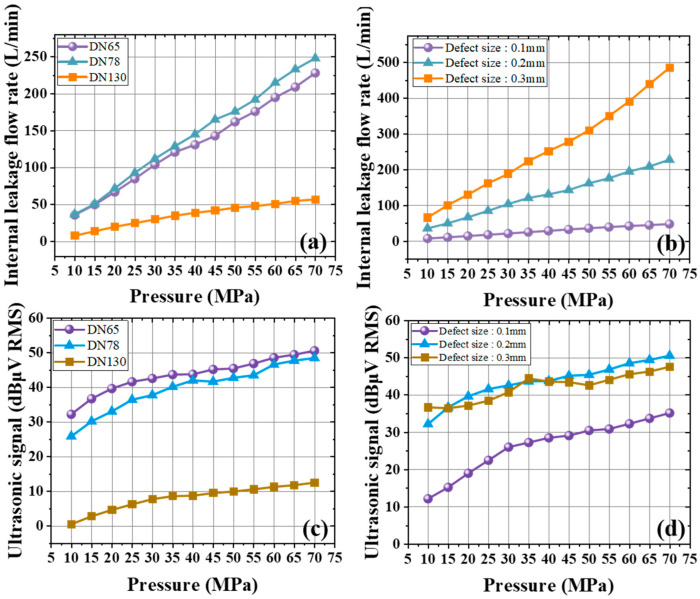
Variation in the internal leakage flow rate of the gate valve in gas when the valve diameter is 65 mm under different pressures with (**a**) different diameters and (**b**) different defect sizes. Variation in the ultrasonic signals at the outlet end of the gate valve in gas for a 0.2 mm defect under different pressures with (**c**) different diameters and (**d**) different defect sizes.

Figure 7a,b shows the internal leakage flow rate under different pressures. With a defect size of 0.2 mm and a pressure range of 10 MPa to 70 MPa, the flow rate for DN65, DN78, and DN130 valves is presented in Figure 7a. As pressure increases, all valve diameters exhibit a rising flow rate: the 65 mm and 78 mm valves grow and the 130 mm valve increases with a more gradual slope, so the ending point of 130 mm valve is much lower than others, about 56 L/min. When the valve diameter is 65 mm and defect sizes are 0.1 mm, 0.2 mm, and 0.3 mm, Figure 7b indicates that flow rates rise with lager pressure, though the degree of increase differs for each defect size. For the group of 0.2 mm and 0.3 mm defects, the strong positive correlation with pressure shows the pronounced effect of high pressure on larger defects. The 0.1 mm defect shows a gentle rise, due to its smaller leakage passage.

Figure 7c,d studies the ultrasonic signals associated with internal leakage at various pressures. With a defect size of 0.2 mm and pressures from 10 MPa to 70 MPa, Figure 7c illustrates the outlet signals for three valve diameters. For the 65 mm and 78 mm valves, ultrasonic signals climb notably as pressure builds, reflecting a pronounced leakage signature. In contrast, the 130 mm valve’s outlet signal also grows but at a lower rate, reaching only 12.4 dBμV RMS at 70 MPa—substantially below the other two diameters—indicating a size-related impact on ultrasonic intensity. When the valve diameter is 65 mm and defect sizes are 0.1 mm, 0.2 mm, and 0.3 mm, Figure 7d demonstrates that outlet ultrasonic signals increase rapidly from 10 MPa to 40 MPa, then level off past 40 MPa, maintaining a consistent trend. Compared with the 0.1 mm defect, the 0.2 mm and 0.3 mm defects produce notably stronger signals as pressure varies, and these two defects are close in magnitude. Thus, larger defects and higher pressures yield higher ultrasonic signal amplitudes.

**Figure 8:**
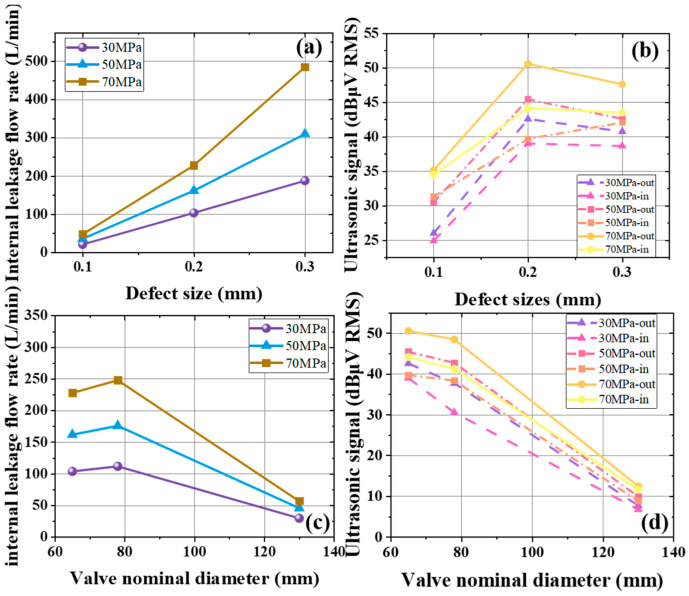
(**a**) Variation in the internal leakage flow rate of the gate valve in gas when the valve diameter is 65 mm under different defect sizes. (**b**) Ultrasonic signals at the inlet and outlet of the gate valve in gas when the valve diameter is 65 mm due to internal leakage under different defect sizes. (**c**) Internal leakage flow rate of gate valves in gas for a 0.2 mm defect with different diameters. (**d**) Ultrasonic signals at the inlet and outlet of the gate valve in gas for a 0.2 mm defect due to internal leakage under different diameters.

Figure 8a presents the internal leakage flow rate for various defect sizes. When the valve diameter is 65 mm and the defect size ranges from 0.1 mm to 0.3 mm, the flow rate under three pressures is shown. Across 30 MPa, 50 MPa, and 70 MPa, there is a strong positive correlation between leakage flow rate and the defect size. As the defect size increases, the flow rate rises sharply. A clear contrast emerges between high pressure (50 MPa) and low pressure (30 MPa). For instance, at a 0.30 mm defect, the flow rate at 50 MPa (310 L/min) is much bigger that at 30 MPa (180 L/min), indicating more severe leakage at larger defect sizes and higher pressures.

Figure 8b illustrate the ultrasonic signals under different defect sizes. With a 65 mm valve diameter at pressures of 30 MPa, 50 MPa, and 70 MPa, Figure 8b compares inlet and outlet ultrasonic signals for the same diameter. At each pressure, the inlet signal also rises from 0.1 mm to 0.2 mm, then decreases after 0.2 mm. Throughout all conditions, the outlet signal remains above the inlet signal. At 0.1 mm and 0.3 mm defects, the inlet–outlet difference remains within about 3 dBμV RMS, which is at 0.2 mm, the outlet signal is stronger than the inlet.

Figure 8c shows internal leakage flow rate across different valve diameters. For a defect size of 0.2 mm and diameters from 65 mm to 130 mm under pressures of 30 MPa, 50 MPa, and 70 MPa, the flow rate initially increases and then decreases with larger diameters. Over the smaller diameter range (60–80 mm), the flow rates at the three pressures exhibit similar differences. As the diameter grows, the flow rates converge under all pressure conditions.

Figure 8d focuses on the ultrasonic signals for different diameters. With a 0.2 mm defect size and diameters from 65 mm to 130 mm at three pressures, Figure 8d compares inlet and outlet signals under the same conditions. Both signals decrease with increasing valve diameter, and the outlet signal remains higher than the inlet signal across all diameters.

**Figure 9:**
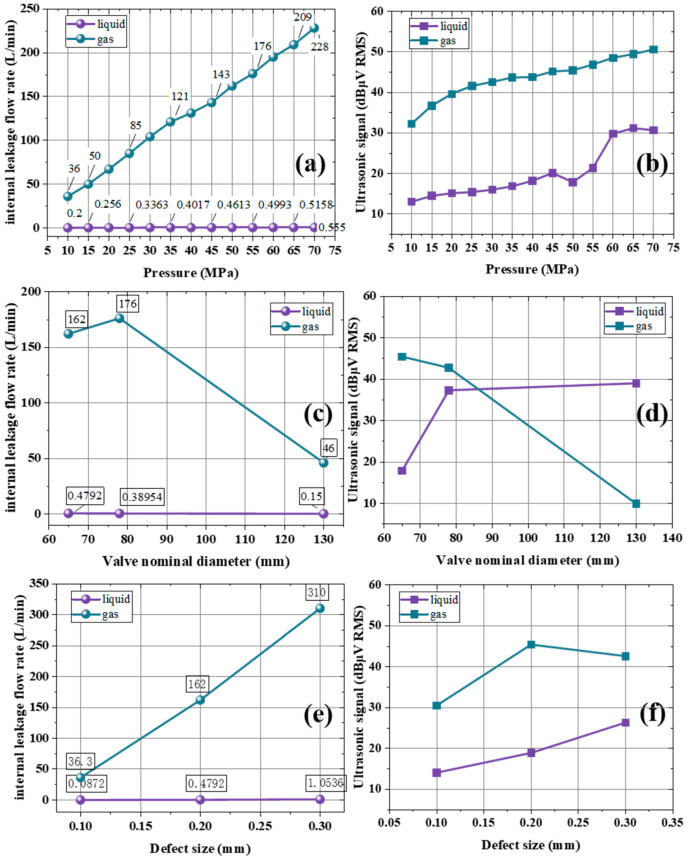
The variation in internal leakage (**a**) flow rate and (**b**) ultrasonic signals in gate valves under different pressures (liquid/gas). And the internal leakage (**c**) flow rate and (**d**) ultrasonic signals of gate valves (liquid/gas) under different nominal diameters. The internal leakage (**e**) flow rate and (**f**) ultrasonic signals of gate valves (liquid/gas) under different defect sizes.

Figure 9a,b compares the internal leakage flowrate and ultrasonic signals of liquid/gas gate valves under different pressures. Figure 9a shows the internal leakage flowrate distribution for a 65 mm diameter gate valve with a defect size of 0.2 mm when the working medium is liquid and gas. Under identical operating conditions, the internal leakage flowrate of the gas medium is much higher than that of the liquid medium. As the pressure increases, the internal leakage flowrate for both media rises, but the growth rate of the gas is noticeably higher than that of the liquid. Figure 9b illustrates the ultrasonic signal at the valve’s leakage outlet under the same conditions but at different pressures. Under identical operating conditions, as the pressure increases, the ultrasonic signal at the outlet also increases for both media. When gas is used as the working medium, the ultrasonic signal at the outlet is substantially larger than that of the liquid medium; as the pressure increases, the difference remains around 20 dBμV RMS. Due to the high compressibility of gas, its internal leakage flowrate and ultrasonic signal vary with changing pressure. Liquid is incompressible so it has less variations in both flowrate and ultrasonic signals. For gas leakage, it shows a higher flowrate corresponds to a stronger ultrasonic signal, indicating a positive correlation.

Figure 9c,d shows the comparison of internal leakage flowrate and ultrasonic signals for liquid/gas gate valves with different nominal diameters. Figure 9c illustrates the internal leakage flowrate distribution of gate valves with a defect size of 0.2 mm at a pressure of 50 MPa for both liquid and gas media and varying valve diameters. Under the same operating conditions, the internal leakage flowrate of both gas and liquid initially increases and then decreases as the valve diameter changes, but the reduction rate of the gas leakage flow is higher than that of the liquid. Figure 9d shows the distribution of the ultrasonic signal at the valve’s leakage outlet under the same conditions but with different valve diameters. Under identical operating conditions, the outlet ultrasonic signals for the two media exhibit distinct trends: as the valve diameter increases, the ultrasonic signal for gas decreases gradually, whereas that of the liquid increases gradually. This phenomenon is closely related to the properties of each medium and the valve size. In summary, because gas is highly compressible, its internal leakage flowrate and ultrasonic signal are extremely sensitive to changes in valve diameter; by contrast, liquid, being nearly incompressible, is only minimally affected. Changing the valve diameter alters the flow space and dynamics of the gas, impacting both its flowrate and ultrasonic signal. Meanwhile, the flow characteristics of liquid remain stable, and the effect of diameter changes is relatively minor.

Figure 9e,f shows the comparison of internal leakage flowrate and ultrasonic signals for liquid/gas gate valves with different defect sizes. Figure 9e presents the internal leakage flowrate distribution for a 65 mm diameter gate valve at a pressure of 50 MPa, with the working medium being either liquid or gas, under different defect sizes. The internal leakage flowrate of the liquid medium remains extremely low with minimal variation, indicating low sensitivity to defect size. In contrast, the internal leakage flowrate of the gas medium increases with larger defect sizes, climbing from 36.3 L/min at 0.1 mm to 310 L/min at 0.3 mm. This pronounced increase indicates that gas leakage flowrate is highly sensitive to defect size: as the defect grows, flow resistance decreases, causing a substantial rise in flowrate. Under the same operating conditions, the gas flowrate is far greater than that of the liquid, and although both media experience an increase in flowrate as defect size grows, the growth rate for gas is substantially higher. Figure 9f shows the ultrasonic signal at the outlet end of the same gate valve under different defect sizes. For the liquid medium, the ultrasonic signal steadily increases as the defect grows, because larger defects generate more ultrasonic energy. For the gas medium, the initial ultrasonic signal is 30.5 dBμV RMS at 0.1 mm), as first rising to about 45 dBμV RMS, then slightly decreasing to 42.6 dBμV RMS, which reveal a rise and fall trend. This pattern reflects the complexity of gas flow characteristics: once the defect reaches a certain size, changes in the flow state led to fluctuations in the ultrasonic signal, yet the overall energy level remains higher than that of the liquid. Under the same operating conditions, the ultrasonic signals at the outlet for both media increase with the defect size, and the gas medium’s ultrasonic signal is always greater than that of the liquid. Overall, during gas leakage, defect size directly affects the flow channel resistance and available flow space, producing dramatic variations in flowrate and corresponding fluctuations in the ultrasonic signal due to the release of flow energy. By contrast, the stable flow characteristics of liquid make it less sensitive to changes in defect size, and thus flowrate and ultrasonic signals rise only gradually.

## 4. Discussion

This study combines theoretical analysis with field experiments to investigate internal leakage in high-pressure valves for liquids and gases under various influencing factors.

Existing studies often focus on detection within relatively narrow domains, such as liquid-only testing or on standard pipelines rather than high-pressure gate valves [3,4,6]. In contrast, our research offers a more comprehensive and integrated approach, considering both liquid and gas condition inside high pressure situation, which is common environment in factory.

Many studies employ methods different from the ultrasonic approach used in our experiments. This method effectively resists signal attenuation in complex flow channels, through time-frequency analysis, leakage signals can be distinguished from background noise with good sensitivity and interference resistance comparing to other existing studies, and others that do use ultrasound often do not cover the full scope encompassed by our study [7,8,9]. Here, we innovatively combine ultrasonic detection with investigations of internal leakage in high-pressure valve systems handling both gas and liquid.

Some prior research limits its leakage tests to simple open/close simulations, showing only the presence or absence of a leak, rather than conducting our more precise defect measurements down to 0.1 mm that reveal both leakage flow rate and defect size. Additionally, existing work often neglects the multifactor analysis we perform, which makes our study more representative of real industrial environments. Our selection of experimental parameters and apparatus also differs from prior studies. We specifically chose valves commonly used in industry, defect sizes based on the smallest precision currently achievable, and high-pressure gate valves for both liquids and gases—widely applicable configurations. By contrast, some existing studies concentrate on specialized experimental environments without incorporating the multifactor, multi-equipment approach adopted here.

We conducted comprehensive on-site experiments and observed certain anomalies that deviated from theoretical expectations, followed by analyses to uncover underlying causes. Such insights can only be discovered through hands-on experimentation, revealing aspects not observable through purely theoretical or simulation-based methods. This provides tangible benefits for industrial applications.

Another discussion is about those anomalies during the research. Experiments include one measurement per condition, anomalies were observed between the experimental data and established theories, which are discussed as follows:

According to current fluid mechanics explanations, as pressure increases, the flow rate through the orifice also increases, and the resulting rise in flow velocity leads to enhanced noise levels. Additionally, for the same orifice size and pressure, a larger orifice diameter results in a greater flow rate. However, in field experiments, several factors influence the results:

Pressure Near Critical Levels: When the pressure approaches 60 MPa to 70 MPa, it nears the valve’s critical pressure value, causing fluctuations in both flow rate and ultrasonic signals.

Defect Size in the 0.3 mm Test Group: The excessive defect size leads to significant external leakage. Consequently, although the internal leakage flow rate increases, the flow velocity does not, resulting in abnormally weakened ultrasonic signals in some experiments with the 0.3 mm defect.

Valve Diameter and Wall Thickness: An increase in valve diameter also leads to an increase in wall thickness, significantly affecting the experimental results in the DN130 tests. As a result, the externally detected ultrasonic signals become abnormally weakened.

These findings elucidate the anomalies observed in the experiments and provide insights into aspects that current theoretical analyses cannot explore experimentally. This contributes significantly to the advancement of academic research in the field.

## 5. Conclusions

This study develops a systematic framework that integrates theoretical modeling with multi-condition experiments to measure the connection with internal leakage in high-pressure gate valves and ultrasonic signals. Experiments include one measurement per condition, which conducted on three valve sizes (DN65, DN78, DN130), three defect sizes (0.1 mm–0.3 mm), two working media (liquid and gas), and seven pressures (10 MPa–70 MPa).

For high pressure liquid gate valve, the internal leakage flow rate of the gate valve rises with increasing pressure. For small-diameter valves, the flow rate accelerates notably as the internal leakage defect grows. The ultrasonic signal at the valve outlet intensifies with higher pressure, remaining relatively stable above 60 MPa. A linear positive correlation exists between the internal leakage flow rate and the defect size; at higher pressures, the leakage flow rate becomes more sensitive to defect growth. Meanwhile, the ultrasonic signal strength increases as the defect size enlarges. Enlarging the nominal diameter typically reduces the flow rate, although DN78 exhibits nonlinear behavior at 70 MPa. The ultrasonic signal at the outlet first goes up and then declines as the valve diameter grows. Throughout the process, the outlet signal remains distinctly higher than the inlet, because turbulence noise and cavitation effects caused by leakage accumulate more at the outlet.

For gas valve, internal gate leakage flow rate increases with rising pressure in all valve diameters; DN65 and DN78 show a more pronounced rate of increase, while DN130 remains relatively moderate. At the outlet, ultrasonic signals for DN65 and DN78 rise with pressure, whereas DN130 exhibits only a slight increase, and smaller diameters generate stronger ultrasonic signals overall. The internal leakage flow rate displays a strong positive correlation with defect size, with larger defects having a more pronounced effect under higher pressure. The ultrasonic signal likewise grows as defects enlarge, showing a marked increase from 0.1 mm to 0.2 mm and then slowing beyond 0.2 mm, particularly at higher pressure. As the valve diameter increases, the internal leakage flow rate initially climbs and then declines, and the ultrasonic signal’s variation with valve diameter generally parallels that of liquid media.

Comparing two media, gas shows stronger and faster responses to rising pressure, while liquid maintains relatively low, stable flow rates. Nonetheless, liquid signals still intensify with defect size, albeit at a lower overall level.

During the research, anomalies were observed between the experimental data and established theories. We have documented anomalies and their underlying mechanisms—previously unaddressed in other studies—whose absence might otherwise lead to discrepancies between theoretical predictions and practical outcomes going unnoticed.

Lastly, the nominal diameters for the valves, the choices of fluid and gas media, and the pressure levels in our experiments are all consistent with common industrial practices, thus broadening the applicability of our results and offering theoretical and experimental support for leak detection in factories. We selected 0.1 mm as our smallest defect size to match the finest precision achievable under current experimental conditions, ensuring that our investigation captures details at the highest possible resolution.

These findings elucidate the anomalies observed in the experiments and provide insights into aspects that current theoretical analyses cannot explore experimentally. They provide support for accurate leakage assessment, enhanced safety management, and the future development of internal leakage quantification models. Through those results, we can support more in-depth research.

## Data Availability

The original contributions presented in this study are included in the article. Further inquiries can be directed to the corresponding author.
